# Supplementary material for: The Complete Chloroplast Genomes of Gynostemma Reveal the Phylogenetic Relationships of Species within the Genus
Source: Genes (Basel). 2023 Apr 17;14(4):929. doi: 10.3390/genes14040929 (PMC10138119; doi:10.3390/genes14040929)
Supplement: Supplementary file 1 [file genes-14-00929-s001.zip › Supplementary Files Figure.pdf]

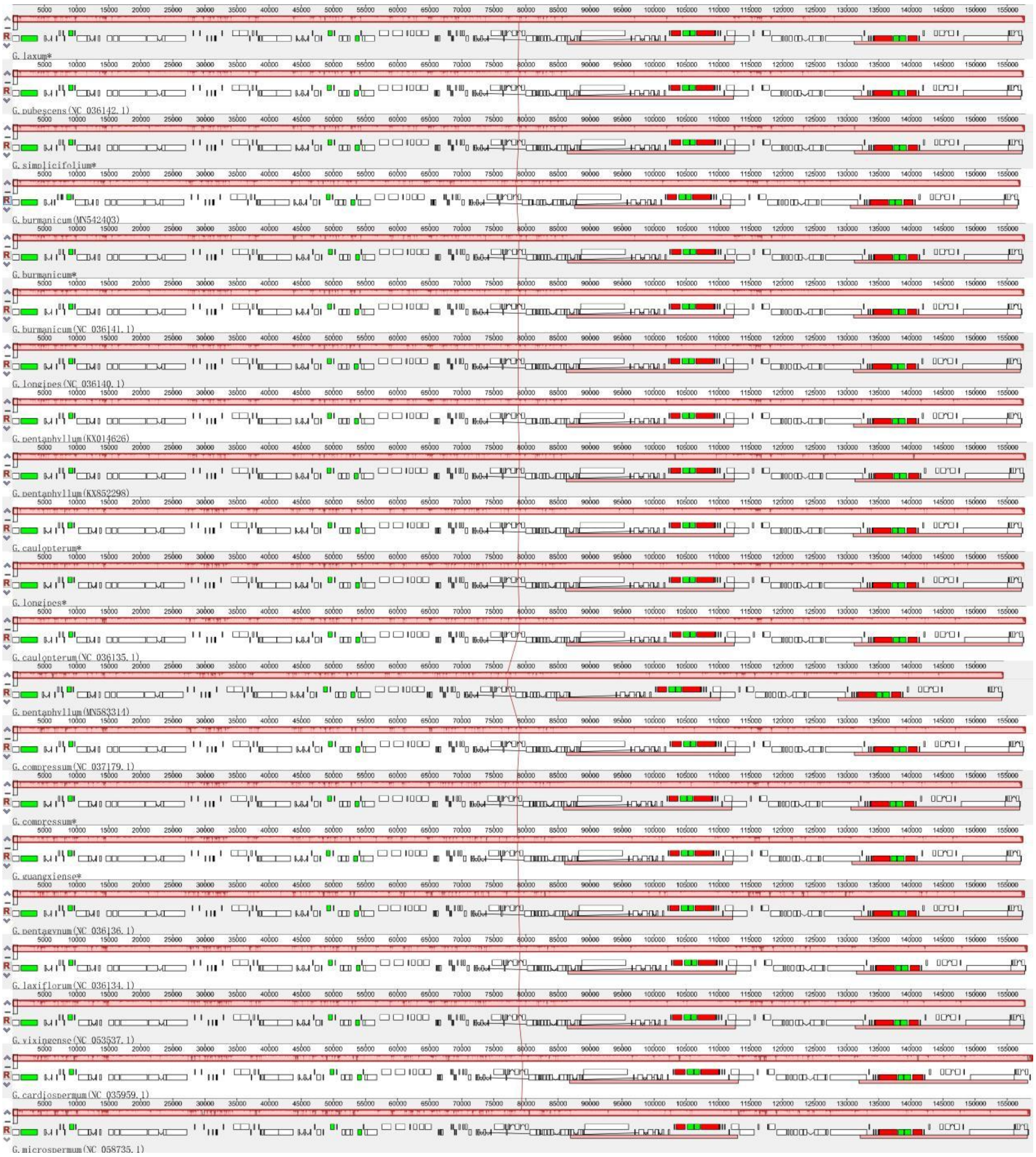

Figure S1. MAUVE alignment of chloroplast genomes of 21 individuals of the genus *Gynostemma*. The differently coloured squares represent different types of genes. Black represents transfer RNAs (tRNAs), and green repersents tRNAs with introns (rRNAs). Red represents ribosomal RNA, while white represents protein-coding genes.

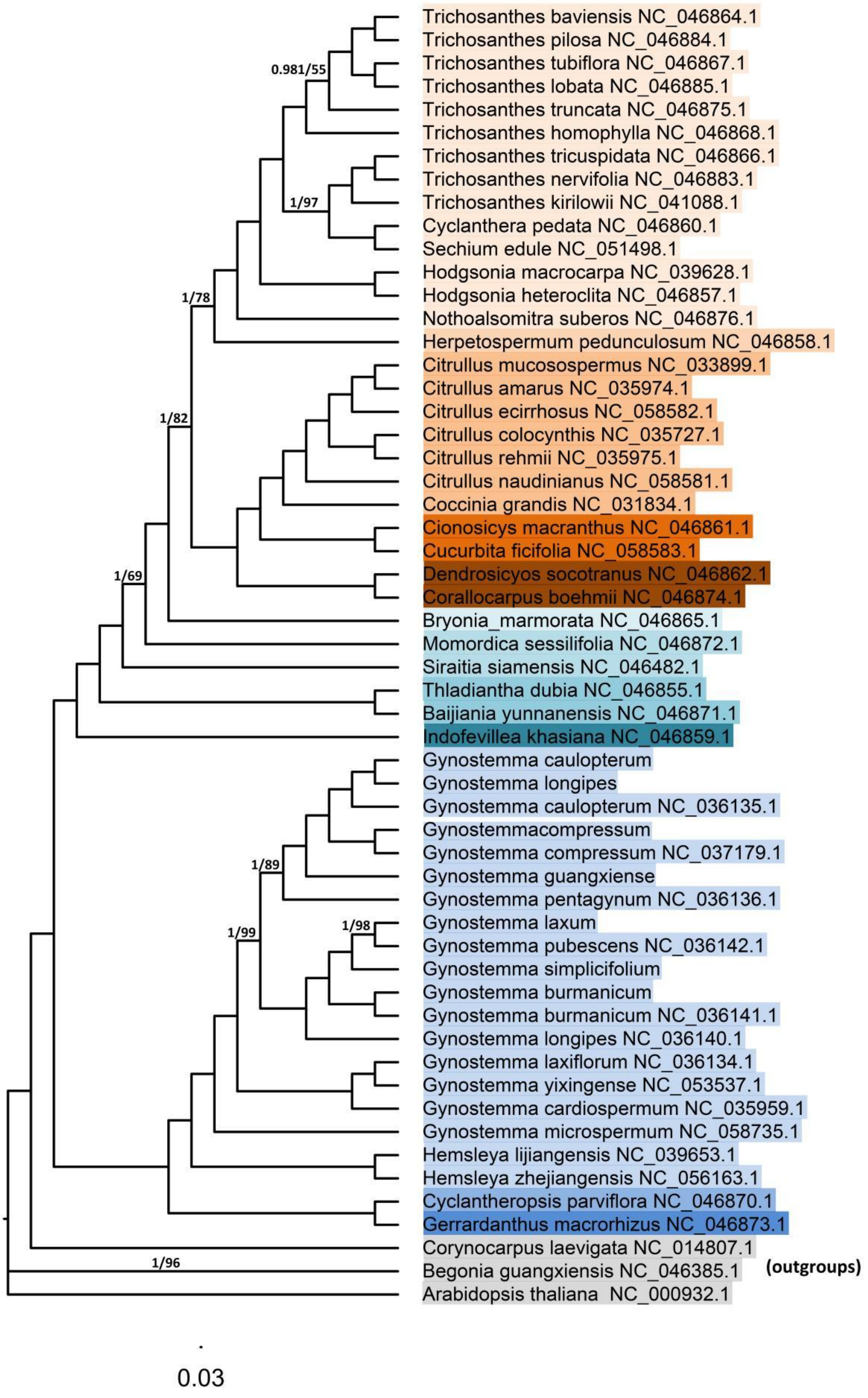

Figure S2. Phylogenetic trees were generated using maximum likelihood (ML) and Bayesian (BI) methods on the basis of 53 *Cucurbitaceae* chloroplast genomic CDS regions.ML trees and BI trees have the same topology.Bayesian posterior probabilities/ML bootstrap values are shown on the nodes and values of 1/100 are unlabelled. Different colours indicate as different tribes according to the APG IV classification system.
